# Structure-Based Engineering Increased the Catalytic Turnover Rate of a Novel Phenazine Prenyltransferase

**DOI:** 10.1371/journal.pone.0048427

**Published:** 2012-10-31

**Authors:** Georg Zocher, Orwah Saleh, Joel B. Heim, Dominik A. Herbst, Lutz Heide, Thilo Stehle

**Affiliations:** 1 Interfaculty Institute of Biochemistry, Eberhard Karls University Tübingen, Tübingen, Germany; 2 Pharmaceutical Institute, Eberhard Karls University Tübingen, Tübingen, Germany; 3 Department of Pediatrics, Vanderbilt University School of Medicine, Nashville, Tennessee, United States of America; University of Michigan, United States of America

## Abstract

Prenyltransferases (PTs) catalyze the regioselective transfer of prenyl moieties onto aromatic substrates in biosynthetic pathways of microbial secondary metabolites. Therefore, these enzymes contribute to the chemical diversity of natural products. Prenylation is frequently essential for the pharmacological properties of these metabolites, including their antibiotic and antitumor activities. Recently, the first phenazine PTs, termed EpzP and PpzP, were isolated and biochemically characterized. The two enzymes play a central role in the biosynthesis of endophenazines by catalyzing the regiospecific prenylation of 5,10-dihydrophenazine-1-carboxylic acid (dhPCA) in the secondary metabolism of two different *Streptomyces* strains. Here we report crystal structures of EpzP in its unliganded state as well as bound to *S*-thiolodiphosphate (SPP), thus defining the first three-dimensional structures for any phenazine PT. A model of a ternary complex resulted from *in silico* modeling of dhPCA and site-directed mutagenesis. The structural analysis provides detailed insight into the likely mechanism of phenazine prenylation. The catalytic mechanism suggested by the structure identifies amino acids that are required for catalysis. Inspection of the structures and the model of the ternary complex furthermore allowed us to rationally engineer EpzP variants with up to 14-fold higher catalytic reaction rate compared to the wild-type enzyme. This study therefore provides a solid foundation for additional enzyme modifications that should result in efficient, tailor-made biocatalysts for phenazines production.

## Introduction

The transfer of prenyl entities is a common enzymatic reaction found in many primary and secondary metabolic processes, for example during terpenoid biosynthesis, attachment of prenyl anchors to proteins, the biosynthesis of lipoquinones, or the production of prenylated secondary metabolites. [1], [2], [3] All of these reactions are catalyzed by prenyltransferases (PTs). A new superfamily of PTs, the soluble aromatic PT family, was identified with the discovery of two PTs involved in the biosynthetic pathway of secondary metabolites, [4], [5], [6] the enzymes CloQ and NphB from *Streptomyces*. The members of the soluble aromatic PT family are found in certain taxa of bacteria and fungi [7], [8] and catalyzes prenylation reactions of diverse aromatic substrates in secondary metabolism. The PT EpzP from *Streptomyces cinnamonensis* DSM 1042 reported here is a member of a new type of bacterial prenyltransferases, as it catalysis the prenylation of a phenazine substrate. Previously described examples of PTs are the fungal enzymes FgaPT2, [9] FtmPT1, [10] CdpNPT [11] and AnaPT [12] and the bacterial proteins NphB, [5] Fnq26, [13] NovQ, [14] Fur7, [15] and CymD. [16] Crystal structure determinations of NphB, [5] CloQ, [17] FgaPT2 [18] and FtmPT1 [19] revealed that all four enzymes share a unique PT-barrel fold. The soluble aromatic PTs have been termed ABBA PTs, [8] due to the presence of five repetitive αββα-motifs that assemble into a ten-stranded antiparallel β-barrel. Furthermore, structures of NphB, [5] FgaPT2 [18] and FtmPT1 [19] in complex with substrates and substrate analogs established the reaction mechanism, which is based on a Friedel-Crafts alkylation. In the first step, the prenyl diphosphate is bound to the enzyme and an electrophilic carbocation is generated, which subsequently attacks the nucleophilic aromatic substrate. In a second step, the resulting Wheland-complex is deprotonated and the product is released from the enzyme. Such a sequential order of substrate binding was determined for NphB [20] and likely also occurs in the homologous PTs.

By sequence comparison, the superfamily of soluble aromatic PTs can be divided into two families. [7], [17] The CloQ/NphB family catalyzes the prenylation of phenols, naphthalenes and phenazines, while the DMATS/CymD family catalyzes the prenylation of indole derivatives. Members of both families are found in eubacteria and fungi. The family of CloQ/NphB enzymes comprises both magnesium-independent PTs (e.g. CloQ) and magnesium-dependent PTs (e.g. NphB). Detailed insight into the catalytic mechanism of indole prenylation by FgaPT2 [18] and FtmPT1 [19] is available, where regioselectivity of the reaction is achieved by a modifiable indole binding site inside the β-barrel. By contrast, knowledge about mechanistic aspects of the prenylation of phenols, naphthalenes and phenazines by CloQ/NphB PTs remains limited, in part because no structural data of such a PT in complex with both of its genuine substrates are available. Although a structure of the magnesium-dependent enzyme NphB in complex with geranyl diphosphate (GPP) and the non-natural substrate 1,6-dihydroxy naphthalene revealed insight into naphthalene prenylation, [5] a more sophisticated understanding of the prenylation reaction, and especially of the determinants of substrate selectivity, regioselectivity and turnover rates, is desired in the group of CloQ/NphB enzymes, in order to fully exploit their large potential as biocatalysts.

**Figure 1:**
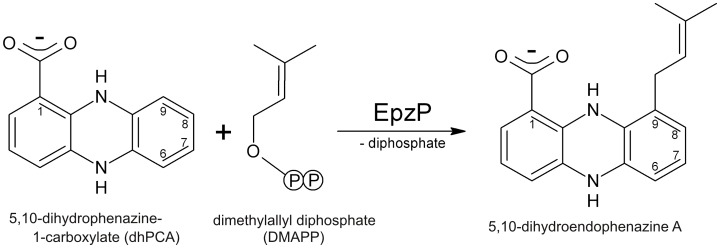
EpzP and PpzP catalyze the regioselective C-prenylation of 5,10-dihydrophenazine-1-carboxylate (dhPCA) to yield 5,10-dihydroendophenazine A.

Recently, the first phenazine PT, PpzP, [21] was identified in *Streptomyces annulatus* 9663 and classified as a member of the CloQ/NphB group. [7] Gene inactivation as well as biochemical studies showed that PpzP catalyzes the regioselective prenylation at the C9 position of 5,10-dihydrophenazine-1-carboxylate (dhPCA) with the use of dimethylallyl diphosphate (DMAPP), yielding the product 5,10-dihydroendophenazine A (Figure 1). [21] Natural phenazine products are secondary metabolites isolated mainly from *Streptomyces* and *Pseudomonas,* and their pharmacological properties include broad-spectrum antibiotic, antitumor, antimalaria and antiparasitic activities. [22], [23] Several phenazine compounds derived from *Streptomyces* strains feature an *N*- or a *C*-prenylated side chain. Prenylation of phenazines contributes to biological diversity [24] and results in a wide range of biological activity. [23] For example, the *N*-prenylated phenazines benthocyanin A–C act as radical scavengers and inhibit lipid peroxidation in rat microsomes, [25], [26] whereas the *N*-prenylated phenazinomycin has antitumor activity against murine tumors and cytotoxic activity against human tumor cells. [27] Antimicrobial activities have been reported by agar plate diffusion assays for the *C*-isoprenylated compounds endophenazine A and C, and it has been shown that these compounds are active against seven out of eight tested Gram-positive bacteria and some fungi. [28].

The phenazine PT EpzP [29] was discovered in *Streptomyces cinnamonensis* DSM 1042. The phenazine biosynthetic gene cluster in this strain is divided into two different chromosomal loci, and previous studies revealed that isoprenoid building blocks utilized by PTs are derived mostly via the mevalonate pathway, but are additionally synthesized via the methyl-erythritol phosphate MEP pathway. [30] Biochemical characterization showed that EpzP [29] catalyzes a prenylation reaction that is identical to that described for PpzP, albeit with substantially lower turnover rates. [21], [29] We have now determined the crystal structure of EpzP from *Streptomyces cinnamonensis* DSM 1042, which is the first structure of a phenazine PT. We report structures of the enzyme in its native state as well as in complex with *S*-thiolodiphosphate (SPP). *In silico* docking of the aromatic substrate dhPCA provides insight into a likely catalytic mechanism, which is also supported by extensive site-directed mutagenesis experiments. Furthermore, based on our structural data and a comparison with PzpP we were able to engineer EpzP to yield a 14-fold higher turnover-rate compared to the wild-type enzyme with the introduction of a single point mutation.

## Methods

### Protein Expression and Purification

Expression of wildtype EpzP from *Streptomyces cinnamonensis* DSM 1042 and mutant proteins was performed using an EpzP-pET28a construct. For protein production, *E. coli* Rosetta2 cells harboring the EpzP plasmid were grown at 37°C and 120 rpm in Luria-Bertani medium containing kanamycin (50 µg/mL) and chloramphenicol (34 µg/mL). 30 min before cell density reached OD600 of 0.6 the temperature was reduced to 20°C. Isopropyl-β-D-thiogalactopyranoside was added to a final concentration of 0.5 mM to start EpzP production. Cells were harvested after 16 h by centrifugation (5440*g, 10 min, 4°C). Cells were resuspended in His-A-buffer (20 mM Tris-HCl pH 8.5, 500 mM NaCl) and placed on ice. Cell lysis was performed by sonication. Subsequent centrifugation (25579*g, 30 min, 4°C) yielded supernatant that was passed through a 0.45 µm filter. The protein solution was loaded onto a 5 mL HisTrap FF column (GE Healthcare) followed by extensive washing with His-A-buffer (10 column volumes). A step gradient with His-B-buffer (20 mM Tris-HCl pH 8.5, 500 mM NaCl, 500 mM imidazole) was applied to remove impurities, and pure EpzP was eluted with 30% of His-B-buffer. Fractions containing pure EpzP were pooled and concentrated. For protein used in activity assays, the buffer was exchanged to SEC buffer (HEPES-NaOH pH 7.5, 150 mM NaCl). Protein folding of EpzP and point mutants of EpzP was verified by circular dichroism spectroscopy. For structure determination the His-Tag of EpzP was removed by thrombin cleavage. This was achieved by exchanging the buffer (20 mM Tris pH 8, 200 mM NaCl, 2.5 mM CaCl_2_), adding thrombin (0.5 U per milligram of EpzP) and incubating for 12 h at 4°C. Uncleaved EpzP, as well as the cleaved His_6_-tag were removed by passing the solution through a 5 mL HisTrap FF column (GE Healthcare) coupled with a benzamidine column. The flowthrough was pooled and concentrated for size-exclusion chromatography using SEC buffer and a Superdex S200/16/60 column. The elution volume of EpzP corresponds to a monomeric state of the enzyme. The pure protein was either used for crystallization trials or methylated prior to crystallization. Initial crystallization trials with the wildtype enzyme failed. Therefore, we performed methylation of the protein sample as 19 lysine residues were found in the sequence of EpzP (302 amino acids). Methylation alters the size and mobility of the lysine side chains and can in some cases promote crystallization.

For methylation of lysine residues the protein sample was diluted to 0.4 mg/mL and placed on ice. Freshly prepared dimethylamine-borane (1 M, 20 µL/mg of EpzP) and formaldehyde solution (1 M, 40 µL/mg of EpzP) were added, and the mixture was extensively stirred. The addition of dimethylamine-borane and formaldehyde was repeated after 3 h and incubated over night at 4°C. Afterwards, aggregates were removed by centrifugation (25579*g, 20 min, 4°C), and the supernatant was concentrated and applied to size exclusion chromatography (SEC), as described above. Successful methylation was determined by mass spectroscopy, which showed that 18 out of 19 lysine residues had been methylated in EpzPm. Proper folding of all EpzP proteins was analyzed with circular dichroism spectroscopy (J-720 CD spectrometer, JASCO; wavelength 205–250 nm), using samples with a concentration of 0.3 mg/mL. CD spectra of the EpzP variants did not show any substantial deviation compared to the CD spectrum of the wildtype enzyme and therefore indicate correct folding of all EpzP variants. All protein samples were flash-frozen in liquid nitrogen until crystallization experiments were performed.

### Crystallization and Structure Determination

All crystallization experiments of EpzP were performed by sitting drop vapor diffusion method. Therefore, 600 nL of the protein solution (30 mg/mL) was mixed 1∶1 with the crystallization buffer and placed on a 96-well plate above the reservoir solution (100 µL). Initially, crystals of methylated EpzP (EpzPm-nat) that diffract to 1.33 Å resolution were obtained in crystallization buffer I (200 mM magnesium chloride, 30% (w/v) PEG4000, Tris hydrochloride pH 8.5) at 4°C. A second crystal form of methylated EpzP grew in crystallization buffer II (200 mM (NH_4_)_2_SO_4_, 30% (w/v) PEG2000MME, 100 mM sodium acetate 4.6) at 20°C. To incorporate dimethylallyl S-thiolodiphosphate (DMSPP), these crystals were soaked with 5 mM DMSPP for 30 min at 20°C to give EpzPm-SPP. Cryoprotection was achieved by transferring crystals into the crystallization solution containing 12% (v/v) PEG400. EpzPwt was crystallized at 20°C (EpzPwt). The protein solution (0.6 µL, 25 mg/mL) was mixed 1∶1 with crystallization buffer III (2.4 M (NH_4_)_2_SO_4_, 100 mM HEPES/NaOH pH 7.5, 200 mM sodium acetate, 0.6 µL). Crystals grew to a final size of 200×100×100 µm^3^ within three months. They were then harvested, mounted in loops, and flash-frozen in liquid nitrogen until data collection.

Data sets of EpzPm-SPP, EpzPm-nat and EpzPwt were recorded at beam line X06DA at the Swiss Light Source in Villigen, Switzerland. Data reduction was performed with XDS and XSCALE. [31] Crystals of EpzPwt belong to orthorhombic space group P2_1_2_1_2_1_, and contain a single monomer in the asymmetric unit. Two copies of the protein were found in the asymmetric units of EpzPm-SPP and EpzPm-nat. Protein phases resulted from molecular replacement using a model of CloQ [17] that had been modified with CHAINSAW [32] ^.^ To avoid model bias, the models were either rebuilt using ARP/wARP [33], or simulated annealing was performed in PHENIX. [34] The final models were obtained after several cycles of manual building with COOT, [35] followed by refinement with REFMAC5. [36] Water molecules were placed using COOT:Find_waters. *S*-thiolodiphosphate in EpzPm-SPP, sulfate and PEG molecules in EpzPwt and EpzPm-nat were inserted with COOT and verified after refinement. The final refinement step involved TLS parameterization [37] using a single TLS group per protomer for EpzPwt and multi-group TLS refinement for EpzPm-SPP and EpzPm-nat. The TLS parameters were obtained from the TLSMD server. [38] The geometries of the final models were analyzed with RAMPAGE [39] and SF-CHECK. [32] Figures were generated using POVscript+ [40] and POVRAY (http://povray.org) or pymol. [41].

### Substrate Modeling

For DMSPP modeling, the diphosphate groups of EpzPm-SPP and NphB (PDB code:1ZB6) were superposed and kept as fixed anchors. The dimethylallyl moiety was placed manually to fit the binding mode of GPP in NphB. The coordinates of the substrate dhPCA were obtained from the PRODRG server [42] and used for *in silico* modeling into the structure of EpzPwt by the use of Vina. [43] For docking calculations, all solvent molecules inside the cavity were removed, with the exception of two structurally conserved water molecules (W1 and W2). The side chain atoms were kept in fixed positions, with the exception of R267, which was known to be flexible, and the search box for the ligand docking experiment was extended to cover the entire active site. Conformations that exhibit the lowest binding energy were investigated for meaningful chemical and biochemical properties of the catalyzed prenylation reaction.

### Mutagenesis

Site-directed mutagenesis was performed to generate site-specific mutations on EpzP. To confirm the active site and the docking calculation, mutant primer pairs were designed to generate the appropriate EpzP mutants: R62N_fwd (5′-GAC CTC GAC TGC AAC TTC ACG ATG CTC CCG AAG GGC CTC GAC CCG-3′) and R62N_rev (5′-GAG CAT CGT GAA GTT GCA GTC GAG GTC GTC CGC GCT GCT GCC GGT-3′); R267Q_fwd (5′-C ACC GAG GGC CAG AAC TAC GTC TAC GGC ATC GCG TCG ACG CCG-3′) and R267Q_rev (5′-GTA GAC GTA GTT CTG GCC CTC GGT GTC GTA CGG CGC GCT CTT GAT G-3′); V270F_fwd (5′-GC CGC AAC TAC TTT TAC GGC ATC GCG TCG ACG CCG AAG GGC GAA TAC-3′) and V270F_rev (5′-GC GAT GCC GTA AAA GTA GTT GCG GCC CTC GGT GTC GTA CGG CGC-3′); S236A_fwd (5′-GG TTC ACG TAC GCG GTC ATG ACG CCG GAG CCG CTG GGC CTC-3′) and S236A_rev (5′-GG CGT CAT GAC CGC GTA CGT GAA CCG GTT GAT CTT CGG CGA G-3′); T64Y_fwd (5′-C TGC CGC TTC TAT ATG CTC CCG AAG GGC CTC GAC CCG TAC GCC CGC-3′) and T64Y_rev (5′-CTT CGG GAG CAT ATA GAA GCG GCA GTC GAG GTC GTC CGC GCT GCT GCC-3′); Y287F_fwd (5′-G ATC GCG TCG TTC TAC CAG TGG CAG AAG CGC GTG GAG AAG CTG-3′) and Y287F_rev (5′-G CCA CTG GTA GAA CGA CGC GAT CTT GTG GTA TTC GCC CTT CGG C-3′); A285L_fwd (5′-C CAC AAG ATC CTG TCG TAC TAC CAG TGG CAG AAG CGC GTG GAG AAG-3′) and A285L_rev (5′-G GTA GTA CGA CAG GAT CTT GTG GTA TTC GCC CTT CGG CGT CGA CG-3′); A285Q_fwd (5′-C CAC AAG ATC CAG TCG TAC TAC CAG TGG CAG AAG CGC GTG GAG AAG-3′) and A285Q_rev (5′-G GTA GTA CGA CTG GAT CTT GTG GTA TTC GCC CTT CGG CGT CGA CG-3′);V218G_fwd (5′-G CGC TCG TTC GGC ATC TAC GTC ACC CTG AGC TGG GAC TCG C-3′) and V218G_rev (5′-GT GAC GTA GAT GCC GAA CGA GCG CTC GCA GAA CTT GAG CAG CTC-3′); G272V_fwd (5′-C TAC GTC TAC GTG ATC GCG TCG ACG CCG AAG GGC GAA TAC CAC-3′) and G272V_rev (5′-CGT CGA CGC GAT CAC GTA GAC GTA GTT GCG GCC CTC GGT GTC GTA C-3′); dM65_fwd (5′-GC CGC TTC ACG CTC CCG AAG GGC CTC GAC CCG TAC GCC CGC-3′) and dM65_rev (5′-CC CTT CGG GAG CGT GAA GCG GCA GTC GAG GTC GTC CGC GCT G-3′). The PCR mixture (50 µL) contained dNTP (1 µL, 25 mM), polymerase buffer (5 µL, 200 mM Tris-HCl (pH 8.8), 100 mM (NH_4_)_2_SO_4_, 100 mM KCl, 1 mg/mL BSA, 1% (v/v) Triton X-100, 20 mM MgSO_4_), Pfu DNA Polymerase (1 µL, Fermentas, 2.5 U/µL), EpzP-pET28a-Plasmid (25 ng), primer premix (1 µL, forward and reverse primer, 100 pM) and DMSO (1 µL). The PCR was performed using a typical thermal cycling (1 cycle: 95°C for 5∶00 min, 18 cycles: 95°C for 1∶00 min; 65.7°C for 1∶00 min; 72°C for 14∶00 min; 1 cycle: 72°C for 20∶00 min). The template DNA was digested by adding Dpn I (Fermentas,1 µL, 10 U/µL) and incubated for 2 h at 37°C. Precipitation of DNA was carried out by adding 1.1 volumes of isopropanol and 0.14 volumes of sodium acetate (3M, pH = 5.2). The mixtures were stored for 2 h at −80°C and then centrifuged (10 min, 13000 g, 4°C). The supernatant was removed, the DNA pellet was dried and diluted in 10 µL water. 5 µL of the DNA were applied for transformation in XL10-Gold® Ultracompetent Cells according to the manual (Stratagene). Successful mutagenesis was verified by sequencing. The mutant proteins were expressed and purified as described above. Correct folding of all EpzP mutants was determined by circular dichroism spectroscopy (J-720 CD spectrometer, JASCO).

### Enzyme Assays

In the prenyltransferase assays, product formation was quantified by HPLC analysis using UV detection at 365 nm. The substrate dihydro-PCA was prepared from commercially available PCA by reduction with dithionite. The enzymatic product dihydroendophenazine A is rapidly oxidized and was therefore converted to the more stable endophenazine A by use of sodium persulfate before extraction from the aqueous reaction mixture by ethyl acetate. The enyzme assay (100 µL) contained 100 mM Na-TAPS (pH 7.5) (Sigma), 0.4 mM freshly prepared dhPCA, 1 mM DMAPP, and 5 µg/mL protein. For the preparation of dhPCA, 90 µL of 50 mM freshly dissolved sodium dithionite (Merck) were mixed with 10 µL of 100 mM PCA dissolved in Tris-HCl 1 M, pH 8.0. 4 µL of this mixture was added to the incubation mixture. After incubation for 5 min at 30°C, 10 µL of 100 mM sodium persulfate (Sigma) were added to oxidize dihydro-PCA and dihydroendophenazine A into PCA and endophenazine A, respectively. The mixture was immediately extracted with 100 µL of ethyl acetate/formic acid (975∶25). After vortexing and centrifugation, 75 µL of the organic phase were evaporated. The residue was dissolved in 100 µL of methanol, and 35 µL thereof were analyzed by HPLC-UV and HPLC-MS. The HPLC-UV analysis was carried out using an Eclipse XDB-C18 column (4.6×150 mm, 5 µm; Agilent) at a flow rate of 1 mL/min with a linear gradient from 40 to 100% of solvent B in 20 min (solvent A: water/formic acid (999∶1); solvent B, methanol) and detection at 252 and 365 nm. Additionally, a UV-spectrum from 200 to 400 nm was logged by a photodiode array detector. For quantitative analysis, authentic reference sample of PCA as external standards was used. The HPLC-MS analysis was carried out using a Nucleosil 100-C18 column (3 µm, 100×2 mm) coupled to an ESI mass spectrometer (LC/MSD Ultra Trap System XCT 6330; Agilent Technology). Analysis was carried out at a flow rate of 0.4 mL/min with a linear gradient from 10 to 100% of solvent B in 15 min (solvent A: water/formic acid (999∶1); solvent B: acetonitrile/formic acid (999.4∶0.6)). Detection was carried out at 230, 260, 280, 360, and 435 nm. Electrospray ionization (positive and negative ionization) in Ultra Scan mode with capillary voltage of 3.5 kV and heated capillary temperature of 350°C was used for LC-MS analysis. Enzymatic activities were calculated in nmol product s^−1^ mg^−1^ enzyme. Activities of EpzP variants were expressed relative to the activity of wildtype EpzP, which was set as 1.

## Results

### Structures of EpzP

**Figure 2:**
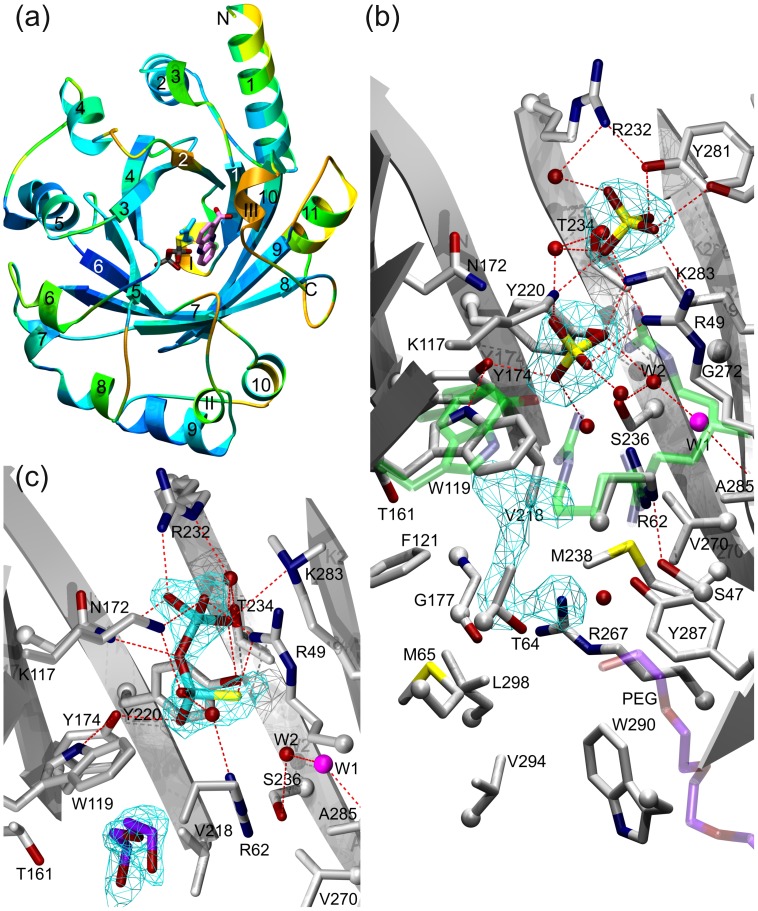
Structures of EpzP. a) Overall structure of EpzPwt reveals an ABBA PT-fold. Secondary structural elements are colored according to their r.m.s. deviations of C_α_-atom positions compared to the most homologous structure of NphB (PDB-Code: 1ZB6) from blue (zero r.m.s.d.) to orange (r.m.s.d. above 3 Å). The substrates dhPCA and DMAPP are modeled and shown in stick representation. **b**) View into the active site of EpzPwt. Side chains which showed a different orientation in EpzPm-nat are depicted in transparent green color. R267 points towards the cavity in EpzPwt and away from it in the EpzPm-nat and EpzPm-SPP structures. Water molecule W1, which is proposed to deprotonate the Wheland complex, is shown in magenta. Two sulfate ions occupy the diphosphate binding site of EpzPwt. The (F_obs_-F_calc_)-omit map (cyan) is shown at σ-level of 3.2 for both sulfate ions and inside the barrel. The remaining electron density in the proposed substrate binding site of EpzPwt could not be explained; it does not fit with any molecule used in downstream purification and crystallization experiments and may represent a molecule inserted into the enzyme during protein production. **c**) The active site of EpzPm-SPP viewed along the same axis. The (F_obs_-F_calc_)-omit map (cyan) is shown at σ-level of 3.0 and clearly reveals the presence of the *S*-thiolodiphosphate moiety (cyan) and a PEG molecule (purple). Hydrogen bonds and hydrophobic interactions are represented with red and green lines, respectively.

Crystallization of unmodified, wild-type EpzP proved initially difficult. However, methylation of the EpzP lysine residues (see Methods) allowed us to grow needle-like crystals (200 mM magnesium chloride, 30% (w/v) PEG4000, Tris hydrochloride pH 8.5) of methylated EpzP (EpzPm). These crystals diffracted to very high resolution of 1.33 Å, belong to the monoclinic space group P2_1_, and were used to assemble a data set for the unliganded protein (EpzPm-nat). Unfortunately, the EpzPm-nat crystals were not suitable for soaking with substrates because the active site is blocked by a symmetry-related protomer from one site, whereas the aromatic binding site is blocked by a PEG molecule (Figure 2b). Therefore, a second crystal form for EpzPm that grew in an acidic crystallization condition (200 mM (NH_4_)_2_SO_4_, 30% (w/v) PEG2000MME, 100 mM sodium acetate 4.6) was used to prepare a complex by soaking with 5 mM of the substrate analog dimethylallyl *S*-thiolodiphosphate (DMSPP). These crystals belonged to the orthorhombic space group P2_1_2_1_2_1_, diffracted to 1.67 Å resolution and were used to determine the structure of EpzP in complex with DMSPP (EpzPm-SPP). Inspection of the electron density map for EpzPm-SPP revealed the presence of a *S*-thiolodiphosphate (SPP) moiety in the diphosphate binding site, implying that hydrolysis of DMSPP had taken place at the acidic pH of 4.5 used during crystallization. Subsequent crystallization screening of non-methylated EpzP eventually yielded a third crystal form (2.4 M (NH_4_)_2_SO_4_, 100 mM HEPES/NaOH pH 7.5, 200 mM sodium acetate) that also belonged to space group P2_1_2_1_2_1_ and diffracted to 1.93 Å resolution. Structure analysis of this crystal from (EpzPwt) and comparison with the two earlier structures confirmed that lysine methylation had not led to structural changes in the protein. Moreover, the EpzPwt structure showed that, in contrast to EpzPm-SPP and EpzPm-nat, the *C*-terminal portion of the enzyme was visible in the electron density and found to seal one entrance of the β-barrel (Figure 2a). All structures of EpzP were refined to the best possible quality and possess low free R-factors, plausible stereochemistry, and exhibit small root-mean-square deviations (r.m.s.d.) from ideal values for bond lengths and bond angles (Table 1). However, as crystals of EpzPwt were not reproducible and other crystal forms could not be obtained, we were unable to prepare a ternary complex. In order to gain insight into the mode of substrate binding, we therefore decided to perform *in silico* based substrate modeling.

**Table 1:** Data collection and refinement statistics^a^.

	EpzPwt	EpzPm-nat	EpzPm-SPP
**Data collection statistics**			
Resolution [Å]	30–1.93	30–1.33	30–1.67
Spacegroup	P2_1_2_1_2_1_	P2_1_	P2_1_2_1_2_1_
Unit cell [Å]	a = 60.6, b = 78.3, c = 83.8	a = 42.1, b = 135.6, c = 48.5, γ = 95.7	a = 42.1, b = 97.0, c = 135.9
No. of reflections			
Measured	144381 (8236)	367267 (9407)	429319 (25178)
Unique	57252 (4072)	115868 (5841)	65469 (4725)
R_meas_ [%]	8.6 (57.3)	6.7 (29.4)	11.9 (59.9)
Completeness [%]	98.6 (94.4)	93.6 (63.7)	99.8 (99.4)
Multiplicity	2.5 (2.0)	3.2 (1.6)	6.6 (5.3)
<I>/<σ(I)>	10.7 (2.0)	13.7 (3.2)	10.7 (2.9)
Wilson B-Factor [Å^2^]	29.5	14.9	20.1
Mosaicity [°]	0.201	0.295	0.212
**Refinement statistics**			
R_cryst_/R_free_ [%]	17.3/19.8	17.9/20.3	18.6/22.4
No. of atoms			
Chain A/B	2315/−	2295/2325	2302/2270
Ions/Water	65/234	39/804	42/639
Average B-Factors [Å^2^]			
Chain A/B	23.7/−	10.2/10.4	15.6/16.6
Ions/Water	43.6/31.1	20.1/18.7	27.7/22.3
Rmsd bond length [Å^2^]	0.0146	0.0117	0.0124
Rmsd bond angle [°]	1.529	1.4507	1.487
Ramachandran angles:			
Favored [%]	97.7	98.0	98.0
Allowed [%]	2.0	2.0	1.6
Outliers [%]	0.3	0.0	0.4

a Values in parentheses are for the highest resolution shell. All data sets were recorded at wavelength λ = 1.0 Å.

### Overall Structure and Comparison to ABBA Prenyl Transferases

EpzP is a soluble, monomeric ABBA PT consisting of 302 amino acids with a molecular mass of 33.2 kDa. The overall structure of EpzP belongs to the PT-fold family [7] and is characterized by ten antiparallel β-strands that assemble into a circular β-barrel. The β-barrel houses a solvent-filled reaction chamber in which the substrates bind and the regioselective Friedel-Crafts prenylation of dhPCA takes place. The β-barrel is surrounded by eleven α-helices that constitute the major fraction of solvent accessible surface of the enzyme (Figure 2a). Additionally, three 3_10_-helices are present in the structure. One of these, the *C*-terminal 3_10_-helix in EpzPwt, seals the reaction chamber of the aromatic binding site of dhPCA.

**Figure 3:**
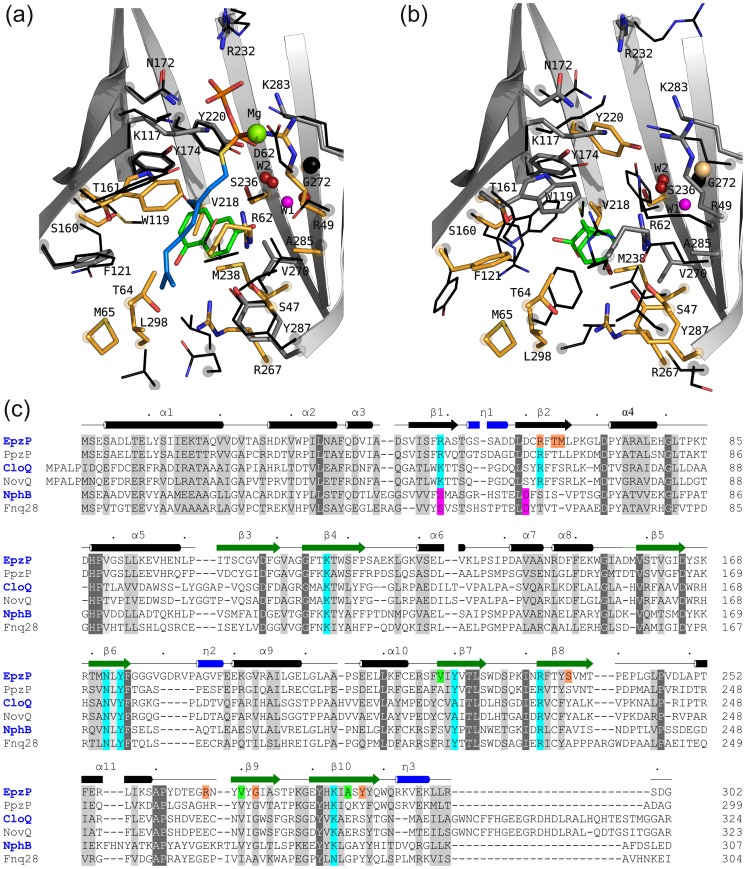
Comparison of EpzP structure and sequence with other PTs of the NphB/CloQ family. a) Superposition of NphB (black lines) and EpzP. **b)** Superposition of CloQ (black lines) with EpzP. The superpositions reveal variability in structure and sequence in the aromatic binding site at the C-terminal part of the enzymes. Side chain residues of EpzP that differ from NphB or CloQ are shown orange, whereas conserved residues are shown grey. The catalytic water molecules W1 and W2 are shown as spheres in magenta and red, respectively. The substrates of NphB and CloQ are shown in stick representation (green). **c)** Structure-based sequence comparison of EpzP, CloQ and NphB and sequence comparison of PpzP, NphB and Fnq28. NphB and Fnq28 are magnesium-dependent enzymes, whereas the others are not. Amino acids of the diphosphate binding site (cyan), mutations that confirm the *in silico* docking model (orange), and mutations that modify the enzymatic turnover (green) are emphasized.

As expected, structural comparisons using DALI [44] and PDBeFold [45] revealed highest similarities to the four known soluble ABBA PT structures. The bacterial PTs NphB from *Streptomyces sp. strain* CL190 [5] and CloQ from *Streptomyces roseochromogenes* [17] are very similar in size (307 and 324 amino acids, respectively). With C_α_-rmsd values of 1.8 Å (288 of 301 aligned amino acids) and 2.2 Å (282 of 301 aligned amino acids), respectively, NphB and CloQ represent the closest structural homologs of EpzP (Figure 2a). The largest deviations among the enzymes occur at the *C*-termini of the enzymes (Figure 3a–c). The dimeric, fungal prenyltransferases FgaPT2 [18] and FtmPT1, [19] both from *Aspergillus fumigatus*, are significantly larger (459 residues for FgaPT2, 464 residues for FtmPT1) but their core regions align well with the ten-stranded antiparallel β-barrel of EpzP, as shown by reasonable C_α_-rmsd values of 3.2 Å for each FgaPT2 and FtmPT1 (231 amino acids were aligned in each case). The additional residues in FgaPT2 and FtmPT1 are found in α-helical and loop regions that connect the core domain. Except for the *C-*termini, these residues do not contribute to the formation of the reaction chamber. Although the β-barrel structures of the NphB/CloQ and DMATS/CymD family of enzymes superimpose well, the architectures of the active centers differ substantially between both families. Since EpzP is a member of the NphB/CloQ family, [7] we will compare the EpzP structure with these two enzymes.

In comparison to EpzP, the aromatic binding site of NphB (Figure 3a and c) is wider and reveals a substantial difference in the geranyl binding cavity. Compared to EpzP, M65 of EpzP is deleted in NphB, which results in a reorientation of strand β2 to facilitate binding of the geranyl entity. A structure comparison of the active center of EpzP with CloQ revealed a substantially smaller cavity for the aromatic substrate of CloQ (Figure 3 and c). The size of cavity in CloQ is restricted mainly by F68, F161, Y233 and W295. All residues are replaced with less voluminous side chains (T64, T161, S236, L298) in EpzP, in agreement with the elongated shape of dhPCA. In summary, the binding site for the diphosphate entity is moderately conserved in structure and sequence, whereas the aromatic binding sites of EpzP, NphB and CloQ differ significantly. A similar observation has been made for the FgaPT2/CymD family, but variations in the aromatic groove are more subtle in that family as substrates share the indole entity as a core feature for substrate binding. [19].

### The Active Center of EpzP

The active site within the β-barrel can be subdivided into the aromatic substrate site and the DMAPP binding site. When the C-terminal 3_10_-helix is defined as the bottom of the β-barrel, the diphosphate binding pocket is found in the upper part of the β-barrel, whereas the aromatic binding site of dhPCA is found in its lower part. The diphosphate binding site is formed by four basic residues (R49, K117, R232 and K283) that compensate for the negative charge of the diphosphate entity (Figure 2b and c). Furthermore, two tyrosines (Y174 and Y220) and an asparagine (N172) contribute to the diphosphate binding as hydrogen bond donors. Additionally, two water-mediated interactions link R62 and T234 to the diphosphate moiety. All basic residues are highly conserved within the family of magnesium-independent PTs. The positively-charged diphosphate binding groove of magnesium-dependent PTs differ at two residues (R49 and R62 for EpzP) (Figure 3a–c). While R62 is not involved in diphosphate binding, R49 is replaced with an aspartate (D51) in NphB. The loss of the positive charge of the R49 side chain in the magnesium-independent PT EpzP is compensated in NphB by a magnesium ion that is coordinated via the aspartate side chain.

The aromatic binding pocket of dhPCA is located opposite to the diphosphate binding site, within a large cavity inside EpzPwt. The pocket is lined with predominantly hydrophobic residues (V47, M65, W119, F121, G177, V218, M238, V270, A285, L298, W290). Only a few polar side chains (S47, T64, T161, Y287, S236) are present, and these could conceivably contribute to substrate binding via a small number of hydrogen bonds (Figure 2b and c). This situation agrees well with the mainly hydrophobic character of dhPCA, a substrate that offers limited options for hydrogen bond formation. Additionally, two positively charged side chains (R62 and R267) are present in this cavity. While R62 contributes to interactions with SPP, R267 does not. Furthermore, the R267 side chain is located in a mostly hydrophobic environment. It is thus tempting to speculate that R267, and perhaps also R62, might interact with the carboxyl function of dhPCA. Interestingly, we found substantial electron density in the cavity of EpzPwt, in an area near the predicted binding site of dhPCA (Figure 2c). The density is indicative of the presence of an ordered compound. Although we were not able to unambiguously identify the nature of this compound, we conclude that it is inserted into the enzyme during protein production, as the shape of the density does not fit with any molecules used in downstream purification and crystallization experiments.

### Conformational Changes in EpzP

In contrast to EpzPwt, which contains a fully ordered *C*-terminus that closes the reaction chamber, the structures of EpzPm-SPP and EpzPm-nat have disordered *C*-termini that cannot be traced beyond residue K292. The lack of an ordered C-terminus renders the aromatic binding site solvent-accessible, and both methylated structures furthermore contain PEG molecules that occupy the cavity (Figure 2b). Interestingly, the opening of the aromatic site is linked to a change in the conformation of R267. In the closed structure of EpzPwt, R267 points towards the active center, whereas in the opened structure of EpzPm-SPP R267 is orientated to the solvent. It is unlikely that this conformational change is related to the methylation of the protein, as K292 (the only lysine residue at the *C*-terminus) is not involved in intramolecular interactions, and no other lysine residue is involved in contact formation with the terminal 3_10_-helix. Furthermore, the flexibility of the C-terminus is confirmed by B-factor analysis, which reveals that the average B-factor for the main chain atoms of residues 292 to 302 is increased by a factor of two. Therefore, we suggest that R267 might trigger a conformational change of EpzP by “scanning” the solvent for the negatively charged substrate and pulling it into the active center resulting in a closed up active site. An important role of R267 for catalysis is furthermore established by *in silico* docking, as well as site-directed mutagenesis.

### Model of Catalysis

Although we were able to incorporate SPP into the binding site of EpzP (EpzPm-SPP), we could not establish a ternary complex of EpzP with its substrate analogue DMSPP and the aromatic substrate dhPCA in our crystallization experiments. Therefore, we modeled the prenyl moiety into the active site and performed *in silico* docking experiments with dhPCA to gain insight into catalysis.

**Figure 4:**
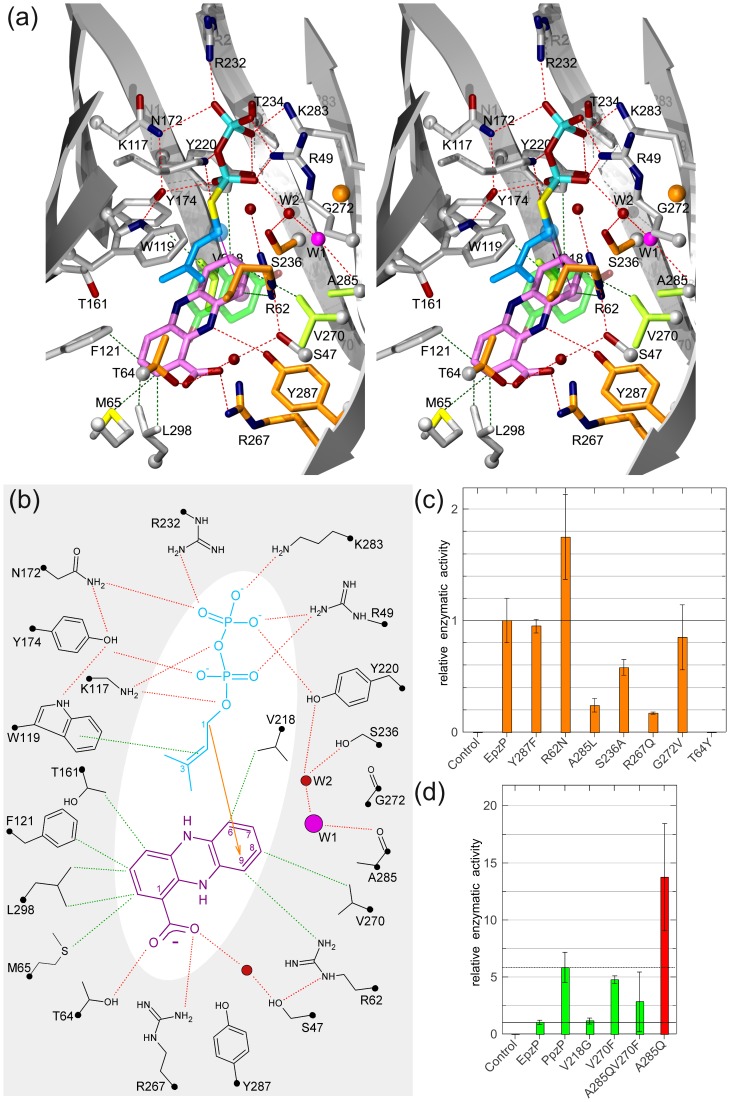
Model of catalysis. **a)** Model of phenazine prenylation based on crystal structures, *in silico* docking and site-directed mutagenesis. The reactions centers of DMAPP (blue) and dhPCA (purple) are emphasized with spheres. Water molecule W1 (magenta) deprotonates the intermediate Wheland complex. EpzP mutants that confirm the model of catalysis or have been engineered to modify turnover-rates are colored orange and green, respectively. 1,6-dihydroxy naphthalene (green), the substrate of NphB, occupies essentially the same binding pocket as dhPCA in EpzP. **b)** Schematic view of substrate binding in EpzP. **c)** and **d**) Relative enzymatic activities of EpzP variants compared to the wild-type EpzP.

Based on the structures of EpzPm-SPP (Figure 2c) and NphB in complex with GPP (see Methods), we modeled DMSPP into the EpzP binding site, utilizing the SPP group as fixed anchors. The dimethyallyl entity faces towards T64 and F121, in such a way that the π-system can interact with the π-orbitals of W119 (Figure 4a and b). Furthermore, F121, T64, R62 and Y174 shield the dimethylallyl moiety. This is comparable with the situation found in the NphB-GPP complex. [5] Here, the geranyl moiety can interact with the aromatic system of Y121 to shield a nascent carbocation during catalysis. Interestingly, W122 in CloQ [17] occupies a different side chain conformation, which would preclude π-π-interaction of the substrate to the aromatic ring system. This difference is most likely a consequence of the absence of sulfate, phosphate or diphosphate groups in the diphosphate binding sites of all structures of CloQ. These ions presumably mimic substrate binding. Comparison of EpzPm-nat, which lacks diphosphate or sulfate in the binding pocket, with EpzPwt and EpzPm-SPP revealed different side chain conformations of W119. In the unbound state, the side chain of W119 is rotated by 180°. Furthermore, residues R62 and R49, which both participate in DMAPP binding, adopt alternative conformations when the anion binding site is not fully occupied (Figure 3b). A substrate-induced fit in EpzP is furthermore supported by the reorientation of Y174 towards the phosphate group during phosphate binding, as this would trigger a side chain flip of W119 as a result of the formation of a hydrogen bond of Y174 to W119 (Figure 2b). The induced fit therefore yields a protected environment that stabilizes the formation of the carbocation during the first step of the catalytic cycle.

The aromatic substrate dhPCA was docked *in silico* into the active site of EpzPwt (see Methods) by the use of Vina. [43] Two structurally conserved water molecules were kept in the dhPCA binding pocket for the docking calculations, whereas all other solvent molecules were removed from the pocket. A planar configuration of dhPCA was used for docking. Structure determination of phenazine complexes in the oxidized and reduced state revealed that the energy barrier between a bent and planar conformation of the tricyclic ring system is low and that dihydrophenazine predominantly adopts a planar conformation. [46] A model of the ternary complex is shown in Figure 4a and b. We selected this model for two reasons. First, it exhibited the lowest binding energy in our docking calculations and revealed meaningful interactions between dhPCA and the protein environment. Secondly, the model is in good agreement with our biochemical data, [29] as it explains the regioselectivity of the catalyzed prenylation reaction. The docking solution is also supported by superpositions of NphB [5] (pdb-code: 1ZB6) and CloQ [17] (pdb-code: 2XLQ) with EpzP, which reveal that dhPCA occupies the same binding pocket as found for the aromatic substrate 1,6-dihydroxy naphthalene in NphB (Fig. 4a) and 2-keto-3-(4-hydroxyphenyl)propanoic acid for CloQ. Furthermore, the distance between the reacting carbon atoms is 4.8 Å and is slightly shorter compared to the distance of 6.2 Å found in the ternary complex of NphB with the non-natural substrate 1,6-dihydroxy naphthalene. [5].

The dhPCA moiety fits tightly into a pocket formed by residues R62, Y174, V218, Y220, S236, R267, V270, Y287 and A285. As a result, the C1-position of DMAPP is oriented towards C9 of dhPCA. An electrophilic attack to C6 of dhPCA seems impossible as steric collisions would preclude the formation of the nascent Wheland complex. It has been shown for CloQ that E281 can act as base to abstract the proton of the sigma complex in order to regain the aromaticity of the product. This has been demonstrated by a point mutation E281Q, which exhibited only 1% of the wild-type activity. [17] Residue A285 of EpzP, which is equivalent to E281 in CloQ, is not able to act as a base. However, two structurally conserved water molecules are located near A285, and both of these are held in place by hydrogen bonds. Water molecule W1 (Figures 4a and b), which we propose acts as base, is fixed via hydrogen bonds to the main chain carbonyl of V270 and the second water molecule, W2, which has tight interactions with the hydroxyl groups of S236 and Y220. Interestingly, both water molecules are also present in the NphB structure, and a similar proton relay for deprotonation has been described in that case. [5] Furthermore, this proton transfer was confirmed in NphB on the basis of quantum mechanics and molecular mechanics simulation. [47].

### Site-directed Mutagenesis of Active-site Residues

In order to provide experimental support for the docking solution, we performed site-directed mutagenesis of residues that are likely to play a role in catalysis. We purified these EpzP variants and determined their catalytic activities as described in a previous study. [29] The substrate dihydro-PCA was prepared from commercially available PCA by reduction with sodium dithionite. The enzymatic product dihydro-endophenazine A is rapidly oxidized and was therefore converted to the more stable endophenazine A by use of sodium persulfate before extraction from the aqueous reaction mixture and HPLC analysis. A summary of the results is depicted in Figures 4c and d. In these figures, reaction velocities observed under catalysis of the different EpzP variants are expressed relative to the reaction velocity under catalysis of wildtype EpzP (0.93 nmol product • s^−1^ • mg^−1^ enzyme). All mutants were purified as soluble proteins and are properly folded as analyzed by CD spectroscopy.

The DMAPP binding site of indole prenyltransferases is highly conserved, and substrate specificity and regioselectivity for the prenylation reaction is obtained via a modifiable reaction chamber at the bottom of the β-barrel of fungal indole prenyltransferases. [19] A similar situation is observed for aromatic PTs in the CloQ/NphB family, which includes EpzP. Structure-based sequence alignments of different members in this family revealed no significant sequence diversities within the binding sites for the prenyl diphosphate, with the exception of the above-described differences between magnesium-dependent and magnesium-independent enzymes. Therefore, a single mutant T64Y was prepared to verify the correct modeling of the dimethylallyl entity of DMAPP. Mutation of T64 to tyrosine abrogates enzymatic activity (Figure 4c), as the increased size of the tyrosine side chain would result in a steric collision with the dimethylallyl entity of the substrate DMAPP. The enzyme cannot avoid this collision, as the side chain of tyrosine is not able to adopt different conformations due to steric restrains in its surrounding. Therefore, no detectable turn-over is observed. This result directly supports the placement of DMAPP and also the proposed catalytic mechanism.

Two positively charged side chains, R62 and R267, are found in the dhPCA binding site of EpzPwt. In principle, both could participate in substrate binding by compensating the negative charge of the carboxyl group of dhPCA. Our docking solution for dhPCA suggests that R267 is involved in binding of the carboxylate group, while R62 mostly contributes to surface complementary, as well as to the water-mediated diphosphate stabilization as described above. These observations are in full agreement with site-directed mutagenesis experiments, as we observe a drastic reduction of catalysis when R267Q is introduced in EpzP (Figure 4c). The reaction velocity drops to 17% of that achieved with the wild-type protein (i.e. to 0.16 nmol s^−1^ mg^−1^), which clearly indicates that the substrate is stabilized by interactions with R267 that cannot be replicated with the shorter glutamine side chain of the mutated enzyme. In contrast, the R62N mutation leads to a significant increase of the velocity (1.63 nmol s^−1^ mg^−1^). The positively charged arginine residue faces towards the hydrophobic phenazine ring system. The exchange to asparagine would avoid such an unfavorable situation and additionally retain water-mediated DMAPP binding. The docking suggested substrate stabilization via a weak hydrogen bond (3.3 Å) from dhPCA to the hydroxyl group of Y287. Enzymatic activity of Y287F remained at wild-type level (0.88 nmol s^−1^ mg^−1^), showing that the hydroxyl group of Y287 is not critical for dhPCA binding. R267 thus appears to be sufficient in fixing the carboxyl group of the phenazine substrate. To investigate the suggested proton relay for deprotonation of the Wheland complex, we prepared a S236A mutant, which should reduce the catalytic activity as the conserved water molecule W2 loses one of its hydrogen binding partners. Indeed, we found that the reaction velocity of this mutant is decreased to approximately 50% of the wildtype enzyme (0.54 nmol s^−1^ mg^−1^). Furthermore, we produced a G272V mutant to determine whether the proton transfer over two water molecules is necessary for deprotonation of the σ-complex. This mutation would be expected to displace the water molecule W2, which lies adjacent to W1. The enzymatic activity remained at wildtype level (0.79 nmol s^−1^ mg^−1^) demonstrating that a proton transfer to the diphosphate moiety is not crucial for deprotonation of the Wheland complex. We investigated the binding mode of the catalytic water W1 to design point mutations which would exclusively displace this water molecule. However, this is not possible as W1 is fixed exclusively by the contact to W2 and a main chain interaction with the carbonyl of V270 (Figure 4c). Next, we prepared EpzP variant A285L. Although, a repulsion of the water molecule W1 and the leucine residue of mutation A285L is possible, which might result in a conformation unfavored for deprotonation, we expected a decrease of catalytic activity of A285L mainly due to steric collision to dhPCA. Indeed, the replacement of alanine in mutant A285L leads to a decrease of the relative reaction velocity to 24% (0.22 nmol s^−1^ mg^−1^) and therefore supports proper docking of the substrates.

### Structure-based Engineering of EpzP

On the basis of our docking solution and the proposed reaction mechanism, we investigated whether modifications of EpzP can be designed that increase the turnover rate of the enzyme to yield higher catalytic turnover rate for chemoenzymatic synthesis. Examination of the docking solution revealed that dhPCA stabilization might be achieved by introduction of V270F. The aromatic side chain of V270F can adopt a geometry that arranges the phenyl ring of the V270F mutant and the substrate dhPCA in a π-stacking conformation. Indeed, the V270F mutant showed a five-fold increased enzymatic activity (4.43 nmol s^−1^ mg^−1^) compared to the wildtype enzyme (Figure 4d). Remarkably, the introduction of this single point mutation led to approximately the same catalytic reaction rate as found for PpzP (5.42 nmol s^−1^ mg^−1^). EpzP and PpzP catalyze an identical regioselective prenylation of dhPCA. Although the affinity to DMAPP and dhPCA were reported to be similar for both enzymes, [21] ^,^ [29] EpzP exhibits substantially lower turnover rates. To determine the structural differences that could underlie these differences in catalytic reaction rate, we prepared a model of PpzP (57% sequence identity) by mapping the sequence of PpzP onto the structure of EpzPwt using MODELLER. [48] We investigated the cavity of the resulting PCA binding site of the PpzP model and found that replacement of four key residues would suffice to essentially replace the dhPCA cavity of EpzP into that of PpzP. EpzP residues T161, V218, R267 and A285 are replaced with V162, A214, H263 and Q281, respectively, in PpzP. A remarkable difference is the replacement of A285 in EpzP with Q281 in PpzP. Therefore, we decided to prepare point mutations A285Q and A285QV270F to investigate the catalytic activity of these substitutions. Instead of V218A, we prepared EpzP variant V218G as substitution of valine to alanine as found in PpzP is proposed to have minor effects on catalysis due to moderate change in side chain size. On the basis of the *in silico* model, no obvious reason for an enhancement of the reaction velocity could be obtained for V218G variant and indeed, the activity remained nearly unchanged when compared with the wildtype enzyme (1.06 nmol s^−1^ mg^−1^; Figure 4d). The enzymatic activity was not further increased in A285QV270F (3.65 nmol s^−1^ mg^−1^) compared to V270F, which indicates that substitution of two voluminous side chain in close vicinity may lead to steric restriction for dhPCA binding. However, we achieved an approximately 14-fold activity for the A285Q mutant (12.89 nmol s^−1^ mg^−1^) compared to wildtype EpzP (0.93 nmol s^−1^ mg^−1^), and a two-fold enhancement compared to PpzP (5.42 nmol s^−1^ mg^−1^). The structure suggests that this boost in catalysis is achieved by providing additional interactions with water molecule W1, which we identified as the likely catalytic base. These additional interactions would be expected to contribute to the rigidity of the dhPCA cavity. The observed drastic enhancement of the reaction velocity of EpzP-A285Q is remarkable, as nature tends to optimize enzymatic reactions and this mutant enzyme is an even more efficient catalyst than the already optimized enzyme PpzP.

## Conclusion and Summary

Aromatic PTs are valuable enzymatic catalysts as they offer the possibility of a regioselective C-C-, C-N- or C-O- functionalisation [49] of aromatic substrates. A relaxed substrate specificity has been observed for several enzymes of this family, for example NphB, [20] Fnq26 [13] or 7-DMATS. [50] The possibility to design their regio- or chemoselectivity by protein engineering [19] renders them attractive candidates for chemoenzymatic synthesis of bioactive substrates, as demonstrated for the production of prenylated indole derivatives. [51] Here we report the structure of EpzP, a magnesium-independent ABBA prenyltransferase and member of the CloQ/NphB family. This is the first structure for a prenyltransferase that is capable of catalyzing the prenylation of phenazine derivatives, and detailed information about its reaction mechanism broadens the spectrum of aromatic substrates for regioselective prenylations by phenazine PTs as well as all ABBA PTs.

In combination with computational docking, the crystallographic data result in a model for the reaction mechanism that is in good agreement with the observed prenylation reaction and is also supported by the results from site-directed mutagenesis experiments. We have been able to identify amino acids that are crucial for catalysis, as demonstrated by EpzP variants T64Y or R267Q. Furthermore, we provide insight into the reaction mechanism of the phenazine prenylation and identify a catalytic water molecule that could act as a base for the deprotonation of the Wheland complex. EpzP variants S236A, G272V and A285L confirmed that this water molecule (W1) nearby the reaction center is important for catalysis and that the proposed proton relay (W2) is not necessary for the deprotonation of the Wheland complex. Such a deprotonation had already been suggested for the catalytic mechanism of NphB, [5] but lacked experimental verification.

We were able to interpret our model for the catalysis and to use it as a template for the design of EpzP modifications. As the reaction velocity for EpzP is reduced 6-fold compared to that of the homologous enzyme PpzP, we probed the differences between the enzymes by analyzing amino acid substitutions in their active sites. Based on this analysis, we established EpzP variants that significantly increased the reaction rate. This impressive result also serves as further confirmation for our docking calculation, especially as mutation V218G, which was designed on the basis of sequence similarity to PpzP but was predicted to have small effects based on the model, indeed showed no substantial change in turnover rates. In contrast EpzP variant V270F increased catalytic turnover rate to PpzP levels, and the mutation A285Q boosted enzymatic performance, in line with our structural model.

In contrast to other PTs, which revealed relaxed substrate specificity, both phenazine PTs are specific for their substrate. [21], [29] We are confident that our structure/function analysis of EpzP can serve as a template for the interpretation of structural and mechanistic properties of other phenazine PTs, including their regioselectivity, and that it can furthermore provide a pathway for the design of optimized phenazine PTs as well as other ABBA-type PTs. Thus, the data presented here elucidate the basis of enzymatic phenazine prenylation and therefore form a platform from which the synthesis of valuable phenazine products by chemoenzymatic methods can be launched.

### Accession Codes

The coordinates and structure factor amplitudes were deposited in the PDB data base under accession codes 4EE6, 4EE7 and 4EE8 for EpzPm-nat, EpzPm-SPP and EpzPwt, respectively.
